# Bestandsaufnahme der verfügbaren und aktuell eingesetzten Typisierungsmethoden von zoonotischen Erregern am Beispiel von Shiga Toxin-bildenden bzw. enterohämorrhagischen *Escherichia coli* (STEC/EHEC)

**DOI:** 10.1007/s00103-022-03628-6

**Published:** 2022-12-16

**Authors:** Anne Richter, Michael Pietsch, Dag Harmsen, Katharina Juraschek, Christina Lang, Alexander Mellmann, Barbara Middendorf-Bauchart, Matthias Pulz, Sarah Roth, Elisabeth Schuh, Angelika Fruth, Antje Flieger

**Affiliations:** 1 Fachgebiet 11: Bakterielle darmpathogene Erreger und Legionellen, Robert Koch-Institut (RKI), Burgstr. 37, 38855 Wernigerode, Deutschland; 2 Poliklinik für Parodontologie und Zahnerhaltung, Universitätsklinikum Münster, Münster, Deutschland; 3 Abteilung Biologische Sicherheit, Bundesinstitut für Risikobewertung (BfR), Berlin, Deutschland; 4 Institut für Hygiene, Universitätsklinikum Münster, Münster, Deutschland; 5 Niedersächsisches Landesgesundheitsamt, Hannover, Deutschland

**Keywords:** Genomische Surveillance, IGS-Zoo, Molekulare Typisierung, Public Health, Genomsequenzierung (*Whole Genome Sequencing, *WGS), Genomic surveillance, IGS-Zoo, Molecular typing, Public health, Whole genome sequencing

## Abstract

**Einleitung:**

Für eine Verbesserung der Patientenversorgung und die Erhöhung der Lebensmittelsicherheit im Sinne von *One Health* wurden im Rahmen des Projekts „Integrierte Genomische Surveillance von Zoonoseerregern (IGS-Zoo)“ Konzepte für eine sektorübergreifende genomische Surveillance von Shiga Toxin(Stx)-bildenden bzw. enterohämorrhagischen *Escherichia coli* (STEC/EHEC) in Deutschland entwickelt.

**Methoden:**

Mittels Onlineumfrage erfolgte zunächst eine Bestandsaufnahme der aktuell in den Laboratorien im Bereich der staatlichen Untersuchungsämter im Veterinärwesen, der Lebensmittelüberwachung und des Öffentlichen Gesundheitsdienstes der Länder verfügbaren und der tatsächlich angewandten Typisierungsmethoden für diese Erreger.

**Ergebnisse:**

Von 33 Teilnehmenden konnten 26 Fragebögen hinsichtlich STEC/EHEC ausgewertet werden. Pro Jahr werden in den Laboratorien zwischen 10 und 3500 Proben bearbeitet, daraus werden zwischen 3 und 1000 Erregerisolate gewonnen. Die aktuell am häufigsten verwendete Typisierungsmethode ist dabei die Bestimmung der Stx- und Intimin-kodierenden Gene mittels Polymerase-Kettenreaktion (PCR). Genomsequenzierung (*Whole Genome Sequencing,* WGS) ist in 8 staatlichen Laboratorien der Länder etabliert und zusätzlich in 9 Einrichtungen geplant. Als häufigstes Hindernis für eine weiterführende STEC/EHEC-Typisierung wurde angeführt, dass auch bei eindeutigem Nachweis des* stx*-Gens mittels PCR eine Isolierung aus Probenmaterial oftmals nicht erfolgreich ist.

**Diskussion:**

Die Ergebnisse der Befragung sollen dazu beitragen, die im Projekt entwickelten Analyseverfahren bei der Zielgruppe zu integrieren, und zudem aufzeigen, wo Schwerpunkte bei der bedarfsgerechten Entwicklung von entsprechenden Schulungskonzepten gesetzt werden sollen.

## Abstract (en)

**Introduction:**

In order to improve patient care and to increase food safety within the framework of One Health, the project “Integrated Genomic Surveillance of Zoonotic Agents (IGS-Zoo)” aims to develop concepts for a genomic surveillance of Shiga toxin(Stx)-producing and enterohemorrhagic *Escherichia coli* (STEC/EHEC) in Germany.

**Methods:**

An online survey was conducted to assess the currently available and applied STEC/EHEC typing methods in the federal laboratories of veterinary regulation, food control, and public health service.

**Results:**

Twenty-six questionnaires from 33 participants were evaluated with regard to STEC/EHEC. The number of STEC/EHEC-suspected samples that the laboratories process per year ranges between 10 and 3500, and out of these they obtain between 3 and 1000 pathogenic isolates. Currently the most frequently used typing method is the determination of Stx- and intimin-coding genes using polymerase chain reaction (PCR). Whole genome sequencing (WGS) is currently used by eight federal state laboratories, and nine are planning to implement it in the future. The most common obstacle for further typing of STEC/EHEC is that isolation from sample material is often unsuccessful despite apparent PCR detection of the *stx* genes.

**Discussion:**

The results of the survey should facilitate the integration of the analysis methods developed in the project and emphasize the target groups’ individual needs for corresponding training concepts.

## Einleitung

Die Typisierung bakterieller Infektionserreger mittels Genomsequenzierung (*Whole Genome Sequencing,* WGS) stellt eine neue, wichtige Entscheidungsgrundlage zur Überwachung und zur Ausbruchsaufklärung dar. Sie trägt dazu bei, die Entstehung und Ausbreitung dieser Erreger besser zu verstehen und im Gegenzug die Ausbreitung von Infektionen durch spezifische Kontroll- und Interventionsmaßnahmen möglichst schnell einzudämmen. Während einige Länder und Institutionen bereits mit der Implementierung der WGS von Zoonoseerregern begonnen haben, z. B. *PulseNet *der Centers for Disease Control and Prevention (CDC) in den USA [1] und *PulseNet International* des European Centre for Disease Prevention and Control (ECDC; [2]) sowie *Public Health England *im Vereinigten Königreich ([3]; heute* UK Health Security Agency and Office for Health Improvement and Disparities*), fehlte in Deutschland bisher eine vergleichbare systematische und koordinierte nationale Initiative.

Deswegen wird im Rahmen der vom Bundesministerium für Gesundheit (BMG) finanzierten Projekte zur „Integrierten genombasierten Surveillance von zoonotischen Erregern und Erregern mit speziellen Antibiotikaresistenzen“ die gemeinsame Einrichtung von Standardprotokollen und die Umsetzung einer ressortübergreifenden Surveillance (Mensch, Tier, Lebensmittel) von zoonotischen Infektionserregern mittels Genomanalyse in 3 Projekten gefördert:

1. Integrierte genombasierte Surveillance von Salmonellen (GenoSalmSurv),
2. Integrierte Genomische Surveillance von Zoonoseerregern (IGS-Zoo) und
3. Genombasierte Surveillance übertragbarer Colistin- und Carbapenemresistenzen Gram-negativer Infektionserreger (GÜCCI).

Im Rahmen des Projekts IGS-Zoo soll ein Konzept zur genomischen Surveillance von Zoonoseerregern in Deutschland am Beispiel der Shiga Toxin(Stx)-bildenden bzw. enterohämorrhagischen *Escherichia coli *(STEC/EHEC) entwickelt, praktisch erprobt und evaluiert werden. Diese Erreger wurden ausgewählt, da sie als Verursacher von Ausbrüchen mit hohen Morbiditäts- und Mortalitätsraten (wie z. B. der durch den EHEC-Stamm O104:H4 verursachte Ausbruch von hämolytisch-urämischem Syndrom (HUS) im Jahr 2011; [4]) sowie der hohen Virulenz und Persistenz besonders relevant sind [5].

Zur Ermittlung der Ausgangslage wurden im ersten Schritt per Onlineumfrage die aktuell verfügbaren Typisierungsmethoden in den Laboratorien im Bereich der staatlichen Untersuchungsämter im Veterinärwesen, der Lebensmittelüberwachung (Lebensmitteluntersuchungsamt, LUA) und des Öffentlichen Gesundheitsdienstes (ÖGD) der Länder erfragt und in welchem Kontext sie tatsächlich angewandt werden. Um synergistische Effekte der involvierten Projektverbünde im Förderstrang „Zoonotische Infektionskrankheiten und Erreger mit speziellen Resistenzen“ zu nutzen, erfolgte die Umfrage in Kooperation mit den Forschungsverbünden GenoSalmSurv und GÜCCI koordiniert durch das Robert Koch-Institut (RKI). Schwerpunkte der Umfrage waren die Geno- und Phänotypie sowie die WGS-basierte Typisierung. Von besonderer Bedeutung ist es, die Faktoren einer sachgerechten Erregertypisierung zu evaluieren und anschließend harmonisierte Verfahren sowie Schulungskonzepte zielgerichtet für die Adressaten dieser Umfrage zu entwickeln. Außerdem sollen so Ansatzpunkte für eine bessere Verbreitung und Nutzung sowie die Verringerung möglicher Hindernisse der genomischen Surveillance identifiziert werden.

## Methoden

### Aufbau und Zielgruppe der Onlineumfrage

Zusammen mit den beiden anderen Forschungskonsortien GenoSalmSurv und GÜCCI wurden 71 Fragen entworfen und auf 5 Themenfelder verteilt: allgemeine Informationen zur Institution [I], allgemeine und spezielle Fragen zur Phäno- und Genotypie von *Salmonella* (GenoSalmSurv, [II_1_]), allgemeine und spezielle Fragen zur Phäno- und Genotypie von STEC/EHEC (IGS-Zoo; [II_2_]), Fragen zur Ausstattung und Nutzung von WGS [III], Fragen zur bioinformatischen Auswertung [IV] sowie Fragen zu (möglichem) Schulungsbedarf [V]. Die Umfrage bestand zum Großteil aus Auswahlfragen mit vorgegebenen Antwortmöglichkeiten und einem Freitextfeld für sonstige Antworten. Angaben zum Institut und zu bearbeiteten Erregern wurden als Pflichtfragen definiert, um eine projektspezifische Zuordnung zu den jeweiligen Projektverbünden zu gewährleisten. Außerdem wurde eine *Survey Logic *eingebaut, sodass einige Zusatzfragen basierend auf den Antworten vorheriger Fragen freigeschaltet wurden. Der Fragebogen wurde mit Hilfe der Umfragesoftware Voxco programmiert und der Zielgruppe online zur Verfügung gestellt. Zur Teilnahme eingeladen wurden insgesamt 16 Landesinstitutionen des ÖGD, 40 staatliche Untersuchungsämter im Veterinärwesen und der Lebensmittelüberwachung sowie 6 Institutionen des Bundes. Für die Auswertung der Umfrage wurden die Antworten entsprechend für die Weiterverwendung in den verschiedenen Konsortien separiert.

Dieser Bericht enthält die projektspezifische Auswertung für IGS-Zoo. Die Auswertung zum Erregerkomplex *Salmonella* sowie die speziellen Teile zur bioinformatischen Auswertung und zum Schulungsbedarf werden in der Publikation „Bestandsaufnahme der verfügbaren und aktuell eingesetzten Typisierungsmethoden einschließlich genombasierter Verfahren von Zoonoseerregern am Beispiel von *Salmonella enterica*“ dargestellt (siehe auch Beitrag von Pietsch et al. in diesem Heft).

## Ergebnisse

### Teilnehmende Laboratorien und Anlass zur Generierung von Typisierungsdaten

**Figure 1:**
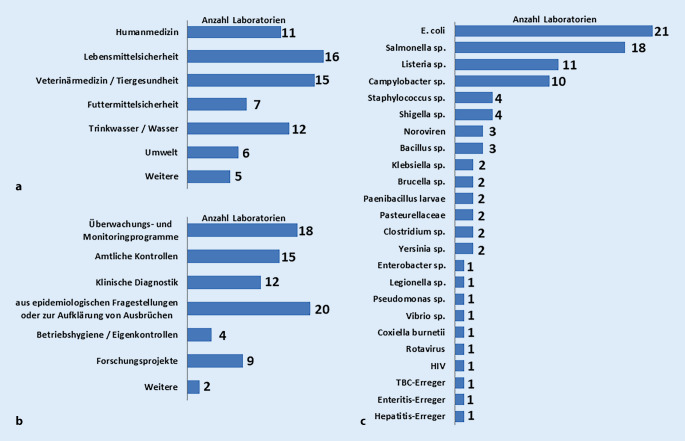

An der gemeinsamen Umfrage der 3 Forschungsverbünde nahmen insgesamt 24 staatliche Laboratorien der Länder, 8 staatliche Laboratorien des Bundes und eine klinische Einrichtung teil. Für das Projekt IGS-Zoo relevant waren davon 26 Fragebögen (22 Landesinstitutionen, 4 Bundesinstitute), von denen 5 Fragebögen nur teilweise ausgefüllt waren. Eine Aufteilung der Laboratorien auf die Sektoren Humanmedizin, Lebensmittelsicherheit, Veterinärmedizin/Tiergesundheit, Futtermittelsicherheit, Trinkwasser/Wasser und Umwelt ist in Abb. 1a dargestellt. Mehrfachnennungen waren möglich. Als weitere Sektoren, in denen die Laboratorien tätig sind, wurden Arzneimittel, Kosmetika, Bedarfsgegenstände, Geologie, Boden, Luft, Infektionsepidemiologie, Hygiene, Pflanzenschutzdienst, Fischereiaufsicht und Tierarzneimittelüberwachung angegeben.

Von den teilnehmenden Laboratorien gaben 19 % (5/26) an, eine Referenzfunktion zu besitzen, davon ist eines ein Referenzlabor nach Verordnung (EG) Nr. 882/2004 bzw. 2017/625, 2 wurden vom BMG als Nationales Referenzzentrum berufen, 2 als Konsiliarlabore und eines fungiert als Referenzlabor nach Tiergesundheitsgesetz. 88 % (23/26) der Laboratorien besitzen eine Akkreditierung, davon sind 70 % (16/23) nach DIN EN ISO/IEC 17025 und 4 % (1/23) nach DIN EN ISO 15189 akkreditiert sowie weitere 26 % (6/23) nach beiden Normen.

Die häufigsten Anlässe, für die in den Laboratorien Typisierungsdaten generiert werden, sind epidemiologische Fragestellungen, Ausbruchsaufklärungen, Überwachungs- und Monitoringprogramme, amtliche Kontrollen oder klinische Diagnostik, gefolgt von Forschungsprojekten und Betriebshygiene/Eigenkontrollen. Weitere Verwendungszwecke sind Erreger-Surveillance und veterinärmedizinische Diagnostik (Abb. 1b).

Auf die Frage nach den 5 häufigsten Erregern, die in den Laboratorien bearbeitet werden, gaben 81 % (21/26) *Escherichia coli*, 69 % (18/26) *Salmonella species (sp.), *42 % (11/26) *Listeria sp.* und 38 % (10/26) *Campylobacter sp.* an. Abb. 1c listet diese und weitere Erreger und zeigt die Anzahl der Laboratorien, in denen sie als häufigste Erreger genannt wurden.

### Anzahl der bearbeiteten Proben und Isolatgewinnung

**Figure 2:**
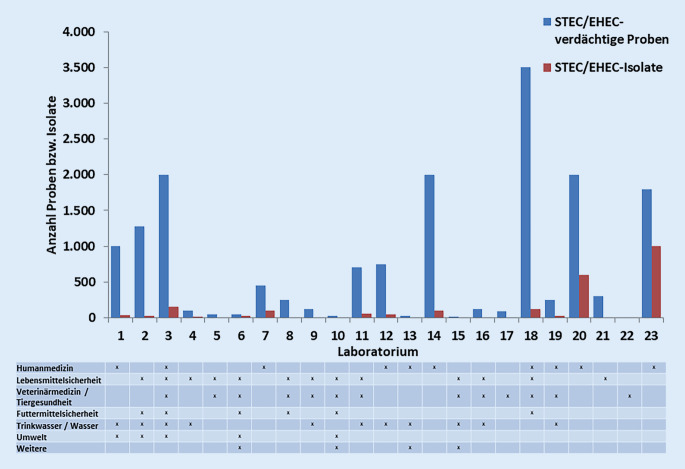

Die Laboratorien wurden gefragt, wie viele STEC/EHEC-verdächtige Proben sie pro Jahr bearbeiten und wie viele Isolate daraus gewonnen werden (Abb. 2). Die Anzahl der STEC/EHEC-verdächtigen Proben, die in den Laboratorien pro Jahr bearbeitet werden, liegt zwischen 10 und 3500. Dabei bearbeiten pro Jahr 31 % (8/26) der Laboratorien unter 100 Proben, 34,6 % (9/26) zwischen 101 und 1000 Proben und 23 % (6/26) über 1000 Proben. 12 % (3/26) der Laboratorien machten hierzu keine Angaben. Die Anzahl der Isolate, die aus den verdächtigen Proben gewonnen werden, ist dabei in allen Sektoren sehr gering; 50 % der Laboratorien gewinnen Isolate aus weniger als 10 % der verdächtigen Proben. 61 % (16/26) der Laboratorien gaben an, dass sie Proben aus Originalmaterial und Isolaten bearbeiten, 27 % (7/26) bearbeiten nur Originalmaterial, 8 % (2/26) nur Isolate und 4 % (1/26) machten keine Angabe.

### Anwendung von Phäno- und Genotypisierungsverfahren

**Figure 3:**
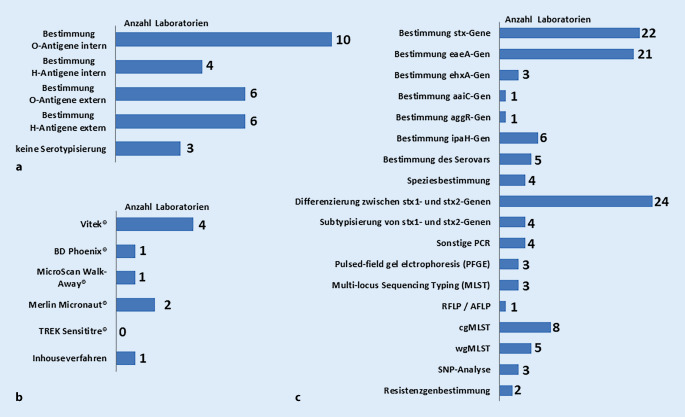

Phänotypische Methoden in der Diagnostik werden von 61 % (16/26) der Laboratorien eingesetzt. Davon machten 15 Laboratorien Angaben, ob und in welcher Form die Serotypisierung angewandt wird, dabei waren Mehrfachnennungen möglich. Zwölf dieser Laboratorien bestimmen O‑Antigene, wobei hiervon 10 eine Bestimmung intern vornehmen und 6 Laboratorien diese extern in Auftrag geben. Neun Laboratorien bestimmen H‑Antigene, davon 4 Laboratorien intern und 6 Laboratorien extern. Drei Laboratorien gaben an, keine Serotypisierung durchzuführen (Abb. 3a). Weitere Verfahren, die in den Laboratorien zur Charakterisierung angewendet werden, sind API (Analytical Profile Index, bioMérieux), Selektivagarplatten, Toxin-ELISA (Enzyme-linked Immunosorbent Assay), Bunte Reihe, Verozelltests und Shiga Toxin Quik Chek (Techlab Inc., Abbott Laboratories).

31 % (8/26) der Laboratorien führen antimikrobielle Empfindlichkeitstests durch. Alle diese Laboratorien gaben an, dafür einen Mikrobouillonverdünnungstest zu benutzen. Als häufigste Testmethode zur antimikrobiellen Empfindlichkeitstestung wird Vitek (bioMérieux) von 50 % (4/8) der Laboratorien genutzt. Weitere Testmethoden sind bei 25 % (2/8) der testenden Laboratorien Merlin Micronaut (Merlin Diagnostika) sowie bei jeweils 12,5 % (1/8) der Laboratorien BD Phoenix (BD Diagnostic Systems), MicroScan Walk-Away (Beckman Coulter) und Inhouseverfahren (Abb. 3b).

Bis auf ein Laboratorium setzen alle molekularbiologische Verfahren zur Charakterisierung der Isolate ein. Alle diese Laboratorien nutzen dazu die Polymerase-Kettenreaktion (PCR) mit Fokus auf *stx*-Gene (88 % [22/25] dieser Laboratorien) und auf das *eaeA*-Gen (Intimin – 84 % [21/25] dieser Laboratorien). Weitere Virulenzgene werden jeweils nur von 12 % (3/25 – *ehxA*), 4 % (1/25 – *aaiC*), 4 % (1/25 – *aggR*) und 24 % (6/25 – *ipaH*) dieser Laboratorien überprüft. Gene zur Bestimmung des Serovars werden von 20 % (5/25) und zur Speziesbestimmung von 16 % (4/25) dieser Laboratorien überprüft.

Molekularbiologische Typisiermethoden zur phylogenetischen Klassifizierung werden von 35 % (9/26) der Laboratorien eingesetzt. Die häufigste dafür angewandte Methode ist das *Core Genome Multilocus Sequence Typing* (cgMLST). Auffällig ist dabei, dass alle teilnehmenden Bundesinstitute (4/4), aber nur 18 % (4/22) der Laboratorien der Länder diese Methode einsetzen. Abb. 3c listet die verschiedenen angewandten Methoden zur Genotypisierung auf.

Sechs der Laboratorien führen einen Vergleich von Isolaten im Rahmen von Ausbruchsuntersuchungen durch. Verwendete Methoden hierfür sind cgMLST, Serotypisierung, SNP(*Single-Nucleotide-Polymorphism*)-Analyse und PFGE (Puls-Feld-Gel-Elektrophorese). 2 Laboratorien (8 %) gaben an, dass die von ihnen generierten Typisierungsdaten bereits in einem juristischen Verfahren verwendet wurden.

Die Laboratorien wurden gefragt, inwieweit die Neufassung von § 13 des Infektionsschutzgesetzes (IfSG) die molekulare Surveillance betreffend in ihrer Einrichtung umgesetzt werden soll. Absatz (3) von § 13 IfSG besagt unter anderem, dass „Einrichtungen des öffentlichen Gesundheitsdienstes, in denen Untersuchungsmaterial und Isolate von Krankheitserregern untersucht werden, verpflichtet sind, Untersuchungsmaterial und Isolate von Krankheitserregern zum Zwecke weiterer Untersuchungen und der Verwahrung an bestimmte Einrichtungen der Spezialdiagnostik abzuliefern (molekulare Surveillance)“. Von einigen Einrichtungen wird dies bereits im Rahmen der Teilnahme am RKI-Netzwerk „Molekulare Surveillance von EHEC“, an einem cgMLST-basierten Pilotprojekt zur molekularen Surveillance in einem Landkreis sowie durch intensivierte Isolatgewinnung und das Deutsche Elektronische Melde- und Informationssystem für den Infektionsschutz (DEMIS) adressiert.

### Hindernisse bei der phänotypischen und genotypischen Charakterisierung von STEC/EHEC sowie Verzögerungen bei der effektiven Typisierung

Als häufigstes Hindernis für eine Typisierung von STEC/EHEC wurde genannt, dass trotz positiver PCR kein *stx*-positiver Stamm aus dem Probenmaterial isoliert werden kann. Weitere Probleme sind das Vorkommen von Mischkulturen, *stx*-Genverlust und dass Stuhlproben eine Vielzahl von Substanzen enthalten können, welche eine Amplifikation mittels PCR inhibieren. Als effektivere *Ad-hoc-*Typisierung wird die Subtypisierung der *stx*-Gene angesehen. Hier wurde als weiteres Hindernis benannt, dass bisher keine kommerziellen PCR-Kits zur Subtypisierung dieser Gene verfügbar sind (siehe Infobox).

### Anwendung von WGS

**Figure 4:**
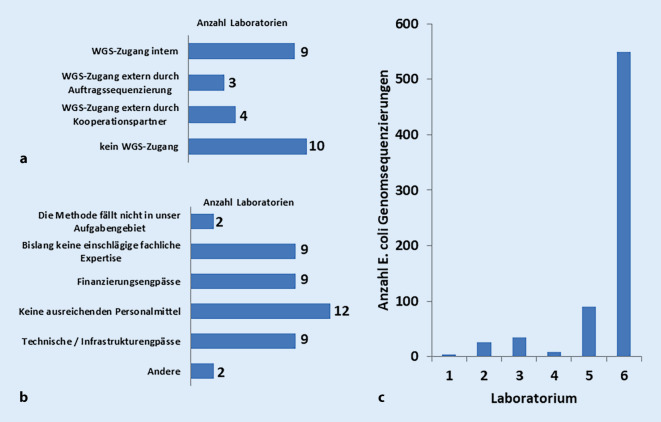

Die Laboratorien wurden gefragt, ob und inwieweit sie Zugang zu WGS besitzen. Die detaillierten Antworten sind in Abb. 4a dargestellt. Es waren Mehrfachnennungen möglich. Insgesamt gaben 12 Laboratorien internen oder externen Zugang zu WGS an. Davon gaben 9 Laboratorien einen internen Zugang an, 3 weitere lediglich externe Nutzungsmöglichkeiten durch Auftragssequenzierung und/oder Kooperationspartner. Zehn Laboratorien gaben an, WGS nicht zu nutzen, 4 Laboratorien gaben keine Antwort. Laboratorien, die WGS nicht oder nur extern nutzen, begründeten dies mit unzureichenden Personalmitteln, fehlender einschlägiger fachlicher Expertise und damit, dass aufgrund von Finanzierungsengpässen oder technischen und Infrastrukturengpässen WGS nicht möglich sei, die Methode nicht ins Aufgabengebiet falle oder derzeit noch etabliert würde bzw. dass es bisher keine Notwendigkeit für die Anwendung von WGS gab (Abb. 4b).

Bei 5 Laboratorien ist die Einführung von WGS geplant. Bei 2 Laboratorien verzögert sich die Einführung aufgrund von Personalengpässen, fehlender Finanzierung und fehlendem Wissenstransfer. Bei 2 weiteren Laboratorien ist wegen fehlender Finanzierung, IT-Infrastruktur, Personalressourcen bzw. technischer und räumlicher Ausstattung lediglich eine externe Einführung von WGS geplant. Ein weiteres Laboratorium plant keine Einführung von WGS, da dafür keine Akkreditierung im humanmedizinischen Bereich vorhanden ist und es zu geringe Probenzahlen gibt.

Die WGS-nutzenden Laboratorien wurden gefragt, ob sich bei der Implementierung Hindernisse ergeben haben. Es wurde von 6 Laboratorien das Fehlen von ausreichenden Personalmitteln angegeben. Je 4 Laboratorien nannten als Hauptgründe für eine lange Implementierungsphase das Fehlen einschlägiger fachlicher oder bioinformatischer Expertise, Finanzierungsengpässe, technische und Infrastrukturengpässe sowie Validierungs- und Normierungsdefizite. Als hinderlich wurden fehlender Austausch und Diskussion von Ergebnissen mit Institutionen unterschiedlicher Zuständigkeiten empfunden. Lediglich ein Laboratorium gab an, dass die Implementierung problemfrei verlief.

Die WGS-nutzenden Laboratorien wurden des Weiteren gefragt, welche Erreger und in welcher Anzahl diese im Jahr 2018 sequenziert wurden. Von den 9 Laboratorien setzten 6 WGS bei *Escherichia coli* ein, dabei lag die Anzahl der Sequenzierungen zwischen 3 und 550 (Abb. 4c). Eine detailliertere Auswertung zur Nutzung von WGS, Bioinformatik und Datenverwaltung erfolgte für alle Konsortien erregerübergreifend ebenfalls im Beitrag von Pietsch et al. in diesem Themenheft, da sich die Antworten der Laboratorien hierzu nicht zwischen den Erregern unterscheiden.

## Diskussion

Durch eine Onlinebefragung wurde in Laboratorien aus dem Bereich der staatlichen Untersuchungsämter im Veterinärwesen, der Lebensmittelüberwachung und des ÖGD der Länder ermittelt, welche Typisierungsmethoden für STEC/EHEC aktuell verfügbar sind und tatsächlich angewandt werden.

Aufgrund der COVID-19-Pandemie ergaben sich bedeutend weniger Rückmeldungen von Teilnehmenden als ursprünglich erwartet. Obwohl die Rückmeldezeit daraufhin verlängert und die Adressaten erneut angeschrieben wurden, blieb die Teilnehmendenzahl insgesamt dennoch sehr gering. Dies hat zur Folge, dass die Daten ggf. nicht repräsentativ für alle Sektoren und Regionen in Deutschland sind. Außerdem spiegeln die Umfrageergebnisse vorrangig die staatlichen Laboratorien der Länder und des Bundes wider, da keine universitären und anderen Forschungslaboratorien, die im Bereich STEC/EHEC tätig sind, an der Umfrage teilnahmen. Da sich je nach Instituts- bzw. Amtsstruktur die Aufteilung der Fachgebiete auf die Sektoren und somit die Anzahl der Adressaten unterscheiden können, sind die absolute Anzahl der potenziell Teilnehmenden und somit die Antwortrate nicht ermittelbar.

Die Befragungsergebnisse bieten jedoch einen Überblick über die genutzten Methoden zur Erregertypisierung sowie mögliche Hindernisse der teilnehmenden Laboratorien. Die aktuell von den meisten Laboratorien genutzte Methode ist die Genotypisierung mittels PCR. Es wird vor allem die Bestimmung der *stx*-Gene sowie des Intimin-Gens durchgeführt. Andere Gene werden jeweils nur von wenigen Laboratorien bestimmt. Phänotypische Methoden zur Charakterisierung von STEC/EHEC werden von knapp 2 Dritteln der teilnehmenden Laboratorien benutzt. Knapp ein Drittel der Laboratorien nutzt außerdem antimikrobielle Empfindlichkeitstests zur weiteren Charakterisierung.

Die weitergehende Charakterisierung der Erreger mittels Serotypisierung und/oder WGS ist für epidemiologische Fragestellungen, insbesondere Ausbruchserkennung und -aufklärungen notwendig. Jedoch wird nur von knapp der Hälfte der teilnehmenden Laboratorien eine Serotypisierung mittels Bestimmung der O‑ oder H‑Antigene durchgeführt und insgesamt ebenfalls nur knapp die Hälfte der Laboratorien haben intern oder extern Zugang zu WGS. In allen teilnehmenden Laboratorien des Bundes wird die WGS genutzt. In den staatlichen Laboratorien der Länder ist die Methode bei knapp einem Viertel der Laboratorien bereits intern einsetzbar, jedoch können weitere Laboratorien externe Möglichkeiten nutzen. Die meisten der Teilnehmenden, die WGS aktuell nicht einsetzen können, planen jedoch, die Methode in ihrem Laboratorium zu etablieren. Lediglich ein Laboratorium schließt dies gegenwärtig aus.

Anhand der Daten der teilnehmenden Laboratorien werden folgende Hindernisse ersichtlich:

In der Hälfte der Laboratorien werden aus weniger als 10 % der verdächtigen Proben tatsächlich Isolate gewonnen. Der geringe Anteil von STEC/EHEC-Isolaten im Verhältnis zu eingegangenen bearbeiteten Proben in einigen Einrichtungen (insbesondere Landeslaboratorien) könnte ebenso darauf zurückzuführen sein, dass hier in größerem Rahmen auch Kontaktpersonenbeprobung von asymptomatischen Personen oder Analysen im Rahmen von Lebensmittel- oder Veterinärmonitoringprogrammen durchgeführt werden. Diese Analysen gehen in der Regel *per se* mit einer geringen Positivrate einher. Jedoch sollte bei Laboratorien mit vorselektiertem Probenaufkommen (wie bei Weiterleitung von auf *stx*-Gene positiv getesteten Proben) der Isolationsanteil höher sein.

Es wurde von mehreren Teilnehmenden als Hindernis genannt, dass trotz positiver PCR keine Isolierung möglich ist. Hier wurden in den letzten Jahren einige ursächliche Faktoren beschrieben. Sowohl die Matrix „Stuhl“ mit dem enthaltenen Mikrobiom als auch bestimmte Transportprozesse und Lagerungseffekte oder das Vorhandensein freier Toxinphagen können die Isolierung der STEC/EHEC-Erreger verhindern bzw. zu falsch-positiven PCR-Ergebnissen führen [6, 7]. Die weitere Analyse und Optimierung von Kultivierungs- und Detektionsbedingungen könnten hier von Bedeutung sein. Forschungsbedarf besteht insbesondere zu Selektivmedien, die eine einfache und schnelle Detektion und Isolierung miteinander koppeln, wodurch zudem die Anzahl durchzuführender Bestätigungstests minimiert werden könnte.

Als Problematik bei der Einführung von WGS wurden vor allem Finanzierungsengpässe und der Mangel an qualifiziertem Personal gesehen. Da WGS-basierte Typisierungsverfahren die Genauigkeit und Effektivität der Analyse von Ausbruchsgeschehen erhöhen, wird es auch im internationalen Kontext zunehmend als Standard gewünscht [8]. Um die Methode erfolgreich in den Laboratorien der Länder zu implementieren, sind eine gezielte Förderung und ein sektorübergreifender Wissens- und Erfahrungsaustausch empfehlenswert.

Im nächsten Schritt von IGS-Zoo ist geplant, eine einheitlich hohe STEC/EHEC-Typisierungsqualität zu etablieren und die entwickelten Analyseverfahren breiter zu integrieren. In diesem Sinne werden Ringversuche durchgeführt und Weiterbildungen organisiert (siehe https://nlga.elearning-home.de). Weiterhin sollen Schwerpunkte bei der bedarfsgerechten Entwicklung von Schulungskonzepten zur Anwendung der Analyseverfahren entwickelt werden.

### Infobox Faktoren, die bei der phänotypischen und genotypischen Charakterisierung von STEC/EHEC hinderlich sind bzw. eine effektive Typisierung verzögern

Antworten der teilnehmenden Laboratorien (*n* = 6) im Freitextfeld auf die Frage, welche Probleme sich bei der phänotypischen und genotypischen Charakterisierung von STEC/EHEC ergeben und eine weitere Typisierung erschweren.

Faktoren, die eine Typisierung verhindern oder verzögern:

- Keine Isolation trotz positiver PCR
- Keine kommerziellen PCR-Kits zur Subtypisierung der *stx*-Gene
- Inhibitoren in Probe, die Amplifikation hemmen
- Mischkulturen
- *stx*-Genverlust
